# Mating systems and protein–protein interactions determine evolutionary rates of primate sperm proteins

**DOI:** 10.1098/rspb.2013.2607

**Published:** 2014-01-22

**Authors:** Julia Schumacher, David Rosenkranz, Holger Herlyn

**Affiliations:** Institute of Anthropology, University of Mainz, Anselm-Franz-von-Bentzel-Weg 7, 55099 Mainz, Germany

**Keywords:** sperm proteins, brain proteins, mating system, sexual selection, functional constraint, sperm competition

## Abstract

To assess the relative impact of functional constraint and post-mating sexual selection on sequence evolution of reproductive proteins, we examined 169 primate sperm proteins. In order to recognize potential genome-wide trends, we additionally analysed a sample of altogether 318 non-reproductive (brain and postsynaptic) proteins. Based on cDNAs of eight primate species (Anthropoidea), we observed that pre-mating sperm proteins engaged in sperm composition and assembly show significantly lower incidence of site-specific positive selection and overall lower non-synonymous to synonymous substitution rates (*d*_N_/*d*_S_) across sites as compared with post-mating sperm proteins involved in capacitation, hyperactivation, acrosome reaction and fertilization. Moreover, database screening revealed overall more intracellular protein interaction partners in pre-mating than in post-mating sperm proteins. Finally, post-mating sperm proteins evolved at significantly higher evolutionary rates than pre-mating sperm and non-reproductive proteins on the branches to multi-male breeding species, while no such increase was observed on the branches to unimale and monogamous species. We conclude that less protein–protein interactions of post-mating sperm proteins account for lowered functional constraint, allowing for stronger impact of post-mating sexual selection, while the opposite holds true for pre-mating sperm proteins. This pattern is particularly strong in multi-male breeding species showing high female promiscuity.

## 1. Introduction

Sexual selection is well known for driving the evolution of diverse male traits in a wide range of taxa, including genital morphology in insects [1], coloration in cichlids [2] as well as sperm mid-piece length and testis size in primates [3,4]. At the molecular level, the size of semen coagulation proteins has been reported to covary with levels of sexual selection in hominoid primates and murine rodents ([5,6]; see also [7]). Other authors observed correlations between evolutionary rates of murine and primate seminal and sperm proteins with species-specific levels of sexual selection as derived from mating systems, testis sizes, number of periovulatory partners or sexual dimorphism of body weight [8–13]. Such associations point to post-mating competition between sperm of different males (sperm competition) as a force enhancing evolutionary rates of male reproductive genes and proteins. However, other forms of post-mating sexual selection, in particular female preference of one spermatozoon over the other (cryptic female choice) [14,15] and conflicts arising from disproportionate costs and benefits of reproductive behaviour between sexes (sexual conflict) [16], can enhance evolutionary rates of male reproductive proteins as well.

At first sight, the above examples suggest that acceleration predominates in the evolution of sperm proteins. But against expectations, many sperm proteins are evolutionarily conserved [17,18]. This can partly be ascribed to additional functions of ‘sperm’ proteins in diverse tissues and organs without relation to reproduction. Accordingly, sperm proteins with testis-specific expression show higher rates of sequence evolution than proteins expressed in testis and other organs or proteins with exclusive expression in non-reproductive tissues [19,20]. Rates of sequence evolution may further be limited by the need to maintain basic protein functions. In *Drosophila*, for example, evolutionary rates of sperm proteins involved in basic functions, such as structure and metabolism, are overall lowered as compared with accessory proteins [17,20]. Still, despite an apparent effect of functional constraint on the evolution of sperm proteins, its impact has not yet been appraised using quantitative measures.

This study aims at assessing the relative impact of both functional constraint and sexual selection on evolutionary rates of functionally distinct sperm proteins. Present analyses are based on 169 human sperm proteins with increased expression in testis or prostate and a clear assignment to one of the following categories: (i) pre-mating sperm proteins that are engaged in sperm composition (cytoskeleton, axoneme and outer dense fibres) or sperm assembly (gene regulation, spermatogenesis and sperm maturation) and (ii) post-mating sperm proteins that prepare (capacitation, hyperactivation and acrosome reaction) or actively participate in fertilization (*zona pellucida*- and egg-binding, gamete recognition, sperm–egg interaction, egg-activation and gamete fusion). The expectation is that species-specific levels of sexual selection may have a stronger impact on evolutionary rates of post-mating proteins, whereas functional constraint may particularly restrict sequence evolution of pre-mating sperm proteins.

Evolutionary rates of primate (anthropoid) sperm proteins were assessed at the cDNA level using the ratio of non-synonymous to synonymous substitution rates (*d*_N_/*d*_S_, also *K*a/*K*s or **ω**). Assuming neutral evolution of synonymous exchanges, *d*_N_/*d*_S_ ratios > 1 stand for selection for more amino acid exchanges than expected under neutrality (positive selection, adaptive evolution). In turn, *d*_N_/*d*_S_ values < 1 can be taken as evidence for negative selection, and hence selection against amino acid exchanges. We quantified levels of functional constraint on the basis of direct and indirect protein interaction partners. The impact of post-mating sexual selection was evaluated by comparing sequence evolution between primate species with higher and lower levels of female promiscuity. Potential associations between *d*_N_/*d*_S_, protein interactions and mating system variation may provide new insights into the mechanisms involved in the evolution of sperm proteins. Furthermore, they may open up new perspectives regarding genes/proteins as targets for diagnosis and treatment of impaired male fertility, development of non-hormonal contraceptives and identification of fertility markers in animal husbandry. In order to verify whether high numbers of direct and indirect protein interactions reflect levels of functional constraint, we compiled and analysed an additional, non-reproductive dataset comprising a total of 318 brain and postsynaptic proteins. The sample of non-reproductive proteins additionally enabled us to control for species-specific differences in demographic history that should affect entire genomes.

## 2. Material and methods

### (a) Functional categorization of sperm proteins

Our sample of 169 sperm proteins was based on three proteomic studies carried out by Ficarro *et al.* [21], Martínez-Heredia *et al.* [22] and Parte *et al.* [23]. The first two investigations were conducted using sperm from normozoospermic men so that all proteins were taken into account. In case of the compilation of Parte *et al.* [23], we only considered proteins from normozoospermic sperm samples and ignored those identified from sperm samples of subfertile individuals. This was done in order to focus on sperm proteins with expression in spermatozoa under physiological conditions. As pleiotropic functions in other tissues might distort analyses addressing the impact of sexual selection on sequence evolution, we included only proteins whose high expression in testis or prostate, as compared with other tissues, had been experimentally verified. Therefore, we screened the EBI Gene Expression Atlas (http://www.ebi.ac.uk/gxa/) for human microarray data and excluded all proteins without consistent information concerning their upregulation in at least one of the search items under consideration (testis, testis germ cell, testis Leydig cell, testis interstitial, testis seminiferous tubule, prostate; state: 15 November 2012). We also excluded proteins, for which no alignment could be compiled containing the aspired species set of eight primates (see below) owing to missing entries, insecure annotation and/or insufficient sequence quality. Based on UniProt gene ontology annotations and original literature, we assigned each of these sperm proteins to one of the following two functional categories:

— Pre-mating sperm proteins (proteins engaged in sperm composition or sperm assembly within the male reproductive tract; see the electronic supplementary material, table S1): the 110 proteins falling into this category are constituents of structural components, such as cytoskeleton (including cytoskeletal calyx and perinuclear theca), axoneme and outer dense fibres. Furthermore, these proteins participate in gene regulation, spermatogenesis or sperm maturation, finally leading to mature spermatozoa ready for ejaculation. The included motor proteins of the dynein complex are involved in the bending of the sperm tail.
— Post-mating sperm proteins (proteins preparing or actively participating in fertilization; see the electronic supplementary material, table S2): the 59 proteins of this category are either involved in post-mating processes increasing sperm motility and priming spermatozoa for sperm–egg interaction (capacitation, hyperactivation and acrosome reaction) or contribute immediately to gamete recognition, sperm–egg interaction, egg-activation and gamete fusion via interaction with female molecules.

### (b) Analyses of sequence evolution

For each of the 169 sperm proteins, we generated a codon-based alignment using the MUSCLE algorithm implemented in the GUIDANCE web-server [24]. Alignments were purified from problematic codon positions using GUIDANCE, leaving only columns with scores higher than 0.93 (default threshold). GUIDANCE alignments were quality checked per eye and newly generated on the basis of manually edited raw alignments when needed. To standardize analyses, we compiled datasets including sequence orthologues of human (*Homo sapiens*, Hsa), common chimpanzee (*Pan troglodytes*, Ptr), western lowland gorilla (*Gorilla gorilla*, Ggo), Sumatran orang-utan (*Pongo abelii*, Pab), northern white-cheeked gibbon (*Nomascus leucogenys*, Nle), Rhesus monkey (*Macaca mulatta*, Mmu), olive baboon (*Papio anubis*, Pan) and white-tufted-ear marmoset (*Callithrix jacchus*, Cja). Coding DNAs were retrieved from ENSEMBL and NCBI databases (for accession numbers, see the electronic supplementary material, tables S3 and S4). Subsequent analyses of sequence evolution were conducted in the maximum-likelihood (ML) framework implemented in the PAML v. 4.4 (phylogenetic analyses by ML) package [25]. The loaded tree represented a basal trifurcation giving rise to Plathyrrhini (New World monkeys), Cercopithecoidea (Old World monkeys) and Hominoidea (apes including humans): (Cja,(Mmu,Pan),(Nle,(Pab,(Ggo,(Ptr,Hsa))))). We specified the F3 × 4 model of codon frequencies and removed sites with ambiguous data (cleandata = 1).

#### (i) Codon-specific analyses

Each of the 169 alignments was tested for the presence of positively selected codon sites employing a likelihood ratio test (LRT) that compares the fit of two beta model versions implemented in Codeml [26]. Both model versions assume a beta distribution of codon sites in the *d*_N_/*d*_S_ interval (0,1). However, while the alternative version (M8) allows for an extra site class under positive selection (*d*_N_/*d*_S_ ≥ 1), *d*_N_/*d*_S_ of this extra site class is fixed at 1 in the null version (M8A). To ensure convergence at global optima, M8 analyses were run thrice with different initial *d*_N_/*d*_S_ values (0.6, 1.2 and 1.6). For LRT, 2*Δ*l was compared with critical values following a 50 : 50 mixture of a point mass at zero and a **χ**^2^ distribution with degrees of freedom (d.f.) equal to the difference in the number of free parameters between M8A and M8 (=1). To reduce the number of false positives, we applied a 1% level of significance (critical value = 5.41) (see the electronic supplementary material, tables S5 and S6).

Subsequently, we analysed if the distribution of proteins with/without candidate sites of positive selection differed between pre- and post-mating sperm proteins using the **χ**^2^-test. Additionally, levels of *d*_N_/*d*_S_ across sites (M8A) were compared between both groups by employing Mann–Whitney *U*-test (two sided). All tests were conducted with SPSS v. 20.0 applying a 5% level of significance and sequential Bonferroni correction for multiple comparisons. We computed 95% confidence intervals (CIs) for each proportion and median on the basis of 100 000 bootstrap replicates using in-house PERL scripts.

#### (ii) Assessing the impact of sexual selection

Effects of post-mating sexual selection on sequence evolution of pre-mating and post-mating sperm proteins were investigated across protein groups considering variant mating systems in the sampled species (see electronic supplementary material, tables S7 and S8; for a compilation of mating systems, see e.g. [27]). In order to recognize potential genome-wide trends, we additionally analysed a sample of 318 non-reproductive proteins (see below; see also the electronic supplementary material, tables S9 and S10).

In the first approach, we ran the free-ratio model (Codeml), which allows *d*_N_/*d*_S_ to vary across branches, on each of the cDNA alignments and compared the estimates for terminal branches to northern white-cheeked gibbon and common chimpanzee. These two species were chosen because they represent the two extremes in the range of mating systems covered by our sample: while common chimpanzees are multi-male breeders with an extraordinarily high number of periovulatory mating partners, northern white-cheeked gibbons are monogamous and extra-pair matings have not been reported ([27,28]; for a compilation of species-specific numbers of periovulatory partners, see [9]).

We additionally ran a branch model that inferred *d*_N_/*d*_S_ values for the terminal branches representing species samples with lower (foreground 1: monogamous gibbon, monogamous human and unimale western lowland gorilla) and higher levels of post-mating sexual selection (foreground 2: chimpanzee and Rhesus monkey). The branches representing white-tufted-ear marmoset and Sumatran orang-utan were sampled into the background instead of foreground 1 owing to frequent extra-pair and -group matings in both species that impair predictions regarding levels of post-mating sexual selection [29–32]. Furthermore, the branch to olive baboon was regarded as a background branch owing to frequent mating of this nominally multi-male breeding species with unimale breeding hamadryas baboon, *Papio hamadryas* [33,34].

Subsequently, we tested gibbon branch, chimpanzee branch, foreground 1 and foreground 2 for different distributions of *d*_N_/*d*_S_ estimates across pre-mating sperm, post-mating sperm and non-reproductive proteins using Kruskal–Wallis rank-sum test (2 d.f., two sided). If Kruskal–Wallis test rejected equality of distribution, we conducted post hoc Mann–Whitney *U*-test on pairs of protein groups (two sided). Kruskal–Wallis and Mann–Whitney *U*-tests were conducted with SPSS v. 20.0 applying a 5% level of significance and sequential Bonferroni correction. Ninety-five per cent CIs of medians were inferred using an in-house PERL script. Whenever short branches impaired the inference of *d*_N_/*d*_S_ estimates for at least one of the terminal branches or foregrounds compared, a protein was excluded from downstream analyses. This procedure led to inclusion of 29 pre-mating sperm, 25 post-mating sperm and 102 non-reproductive proteins when focusing on the branches to common chimpanzee and northern white-cheeked gibbon (see the electronic supplementary material, tables S7, S8 and S10). Comparisons of foregrounds 1 and 2 were based on 44 pre-mating sperm, 43 post-mating sperm and 177 non-reproductive proteins (see the electronic supplementary material, tables S7, S8 and S10).

### (c) Numbers of protein–protein interactions as a proxy of functional constraint

We assessed levels of functional constraint for each of the 169 sperm proteins based on numbers of direct and indirect protein–protein interaction partners (PIP) as taken from 17 out of 25 databases available through PSICQUIC (Proteomics Standard Initiative Common QUery InterfaCe; state 27 May 2013), using human protein IDs as search items (see the electronic supplementary material, tables S1 and S2) and employing the PSICQUIC clustering feature that provides a non-redundant list of interactants. The GeneMANIA, iRefIndex, Interoporc and STRING databases were opted out in order to avoid that assumed (instead of proven) interactions biased our results. For the same reason, we ignored search results with the attributes ‘unspecified method’, ‘predictive text mining’ and/or ‘inferred by curator’ (quotation marks highlight PSICQUIC terminology). Moreover, results without information about the underlying methodology or referring to interactions between human proteins and proteins of other species including pathogens were excluded. Therefore, we also ignored hits from the MPIDB and VirHostNet databases, which focus on interactions with microbes and viruses. Additionally, we avoided to record interactions between proteins and drug-like molecules, and thus excluded the BindingDB and ChEMBL databases. In the electronic supplementary material, tables S5 and S6, we list numbers of interactions per sampled pre-mating and post-mating sperm protein.

We checked for a correlation between the number of direct and indirect protein interaction partners per protein (*n*PIP) and *d*_N_/*d*_S_ across sites (M8A), employing Spearman's rank correlation. Additionally, we investigated whether levels of *d*_N_/*d*_S_ across sites (M8A) differed between 71 sperm proteins with *n*PIP < 10 and 16 sperm proteins with *n*PIP > 100 using Mann–Whitney *U*-test. Finally, we examined whether levels of *n*PIP differed between pre- and post-mating sperm proteins, employing Mann–Whitney *U*-test again. Ninety-five per cent CIs of medians (*d*_N_/*d*_S_, *n*PIP) were inferred from 100 000 bootstrap replicates, each. All analyses were carried out with SPSS v. 20.0 applying a 5% level of significance and sequential Bonferroni correction for multiple testing. An analogous procedure was applied to a set of 318 non-reproductive proteins (see below), thereof 136 with *n*PIP < 10 and 29 with *n*PIP > 100 (see the electronic supplementary material, tables S5, S6 and S10).

### (d) Sample of non-reproductive proteins

In order to (i) recognize potential effects of demography on sequence evolution of sperm proteins and (ii) validate a potential relationship between *n*PIP and sequence evolution, we compiled a sample of non-reproductive proteins, adopting a previous approach [35]. This sample contained 318 human brain and postsynaptic density proteins from proteomic studies of Dumont *et al.* [36] and Bayés *et al.* [37] that showed no upregulation in testis or prostate according to EBI Gene Expression Atlas (search items as described for sperm proteins; state 27 November 2012). The complete set of eight orthologous cDNAs was available for each of the sampled non-reproductive proteins. Accession numbers of cDNAs and data on sequence analyses and *n*PIP are reported in the electronic supplementary material, tables S9 and S10.

## 3. Results

### (a) Differential proportions of site-specific positive selection and levels of *d*_N_/*d*_S_ across codon sites in functionally distinct sperm proteins

**Figure 1.**
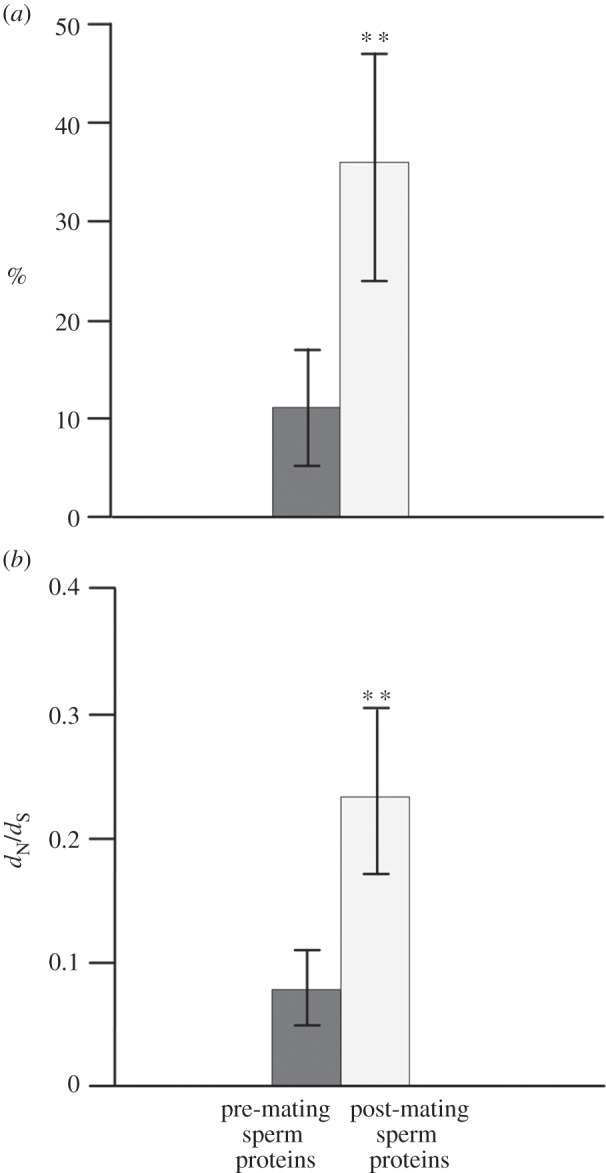
Sequence evolution of functionally distinguished sperm proteins as inferred across eight primate orthologues. (*a*) Group-specific proportions illustrate significantly higher incidence of positively selected codon sites in sperm proteins preparing or actively participating in fertilization (post-mating sperm proteins) than in sperm proteins adopting functions within the male reproductive tract (pre-mating sperm proteins). The presence of positively selected codon sites was assessed at the 1% level of significance applying a LRT (Codeml M8A/M8). (*b*) Levels of *d*_N_/*d*_S_ estimates (medians, M8A) are significantly increased in post-mating sperm proteins relative to pre-mating sperm proteins across the sampled primate orthologues. Vertical bars define 95% CIs calculated from 100 000 bootstrap replicates. Double asterisks (**) highlight support from (*a*) **χ**^2^ and (*b*) Mann–Whitney *U*-test at the 1% level of significance after sequential Bonferroni correction. See the electronic supplementary material, table S11.

At the 1% level of significance, LRT statistics supported the presence of positively selected codon sites for 33 out of 169 cDNA alignments (see the electronic supplementary material, tables S5 and S6), each representing a constant set of eight primate (anthropoid) species. The proportion of alignments including candidate sites of positive selection was markedly higher in post-mating sperm proteins preparing or actively participating in fertilization (36%) than in pre-mating sperm proteins engaged in sperm composition or sperm assembly (11%) (figure 1*a*; electronic supplementary material, table S11). Additionally, median *d*_N_/*d*_S_ values (M8A) pointed to overall enhanced non-synonymous/synonymous substitution rate ratios of post-mating proteins (=0.233) versus pre-mating sperm proteins (=0.077) (figure 1*b*). In line with this, **χ**^2^ and Mann–Whitney *U*-tests provided highly significant support for increased incidence of site-specific positive selection and overall higher *d*_N_/*d*_S_ values (M8A) in post-mating relative to pre-mating sperm proteins (*p* < 0.01, each; electronic supplementary material, table S11).

### (b) Branch-specific *d*_N_/*d*_S_ values against the background of protein-function and mating system variation

**Figure 2.**
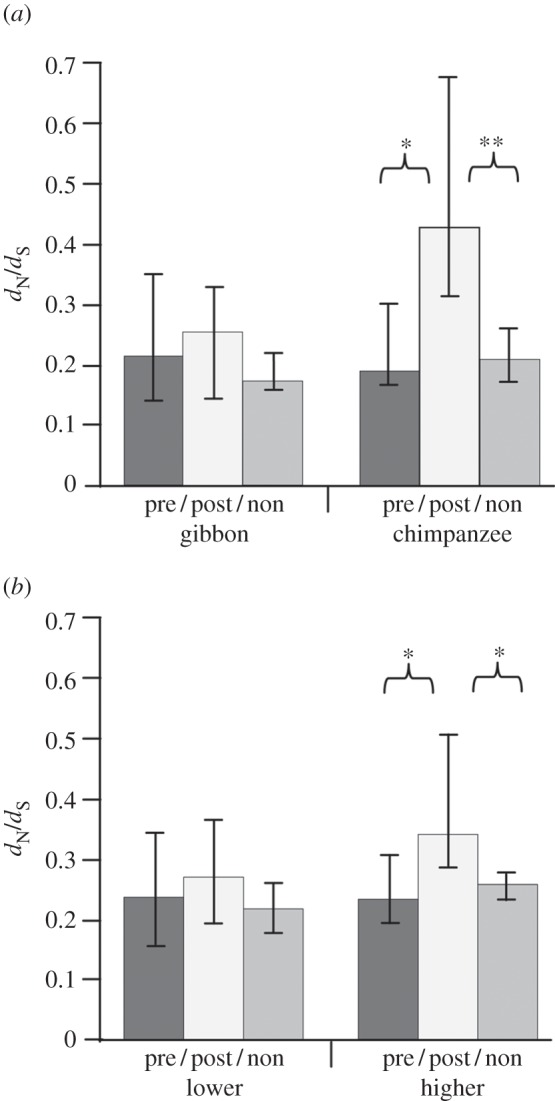
Sequence evolution of pre-mating sperm (pre), post-mating sperm (post) and non-reproductive proteins (non) against the background of variant mating systems in primates. Kruskal–Wallis test supported differential levels of *d*_N_/*d*_S_ across the three distinguished protein classes exclusively for the terminal branches to multi-male breeding species (right panels in (*a*) and (*b*)), but not for the terminal branches representing species with less intense post-mating sexual selection (left panels). Post hoc Mann–Whitney *U*-test provided significant support for increased levels of *d*_N_/*d*_S_ values in post-mating sperm versus pre-mating sperm and non-reproductive proteins for the branches to species with higher sperm competition levels (right panels). The described patterns were reproduced, irrespective of (*a*) confining analyses to monogamous northern white-cheeked gibbon (gibbon) and multi-male breeding common chimpanzee (chimpanzee) or (*b*) taking into account species samples representing lower (northern white-cheeked gibbon, human and western lowland gorilla; lower) and higher levels of female promiscuity (common chimpanzee and Rhesus monkey; higher). Columns and vertical bars define medians and 95% CIs derived from 100 000 bootstrap replicates. Double (**) and single asterisks (*) highlight support from post hoc Mann–Whitney *U*-test at the 1% and 5% level of significance, respectively, after sequential Bonferroni correction. See the electronic supplementary material, tables S12 and S13.

Kruskal–Wallis test rejected equal distribution of *d*_N_/*d*_S_ estimates across pre- and post-mating sperm and non-reproductive proteins for the branch to multi-male breeding common chimpanzee (*p* < 0.05), but not for the branch to monogamous northern white-cheeked gibbon (*p* > 0.05; electronic supplementary material, table S12). Indeed, 95% CIs of median *d*_N_/*d*_S_ estimates were highly overlapping with respect to the gibbon branch. On the contrary, 95% CI of post-mating sperm proteins ranged above the respective intervals of pre-mating and non-reproductive proteins with regard to the chimpanzee branch (figure 2*a*). As far as the chimpanzee branch was concerned, levels of *d*_N_/*d*_S_ estimates were more than twofold higher in post-mating sperm proteins (median *d*_N_/*d*_S_ = 0.428) than in pre-mating sperm (median *d*_N_/*d*_S_ = 0.190) and non-reproductive proteins (median *d*_N_/*d*_S_ = 0.211) (figure 2*a*; electronic supplementary material, table S12). Accordingly, post hoc analyses of the chimpanzee branch provided significant support for different *d*_N_/*d*_S_ levels in post-mating sperm relative to pre-mating sperm proteins (*p* < 0.05) and non-reproductive proteins (*p* < 0.01; Mann–Whitney *U*-test, each). However, no such support was provided when comparing *d*_N_/*d*_S_ estimates of pre-mating sperm and non-reproductive proteins for the chimpanzee branch (*p* > 0.05; Mann–Whitney *U*-test; electronic supplementary material, tables S7, S8, S10 and S13).

These findings could be reproduced when expanding analyses from chimpanzee and gibbon to our species samples representing higher and lower levels of post-mating sexual selection. Thus, test results suggested unequal evolutionary rates of the three distinguished protein classes for the terminal branches to multi-male breeding chimpanzee and Rhesus monkey (*p* < 0.05), but not for the branches to monogamous gibbon and human and unimale breeding gorilla (*p* > 0.05; Kruskal–Wallis test, each; electronic supplementary material, table S12). Moreover, Mann–Whitney *U*-test confirmed significantly higher *d*_N_/*d*_S_ estimates for post-mating sperm proteins (median *d*_N_/*d*_S_ = 0.339) as compared with pre-mating sperm proteins (median *d*_N_/*d*_S_ = 0.233; *p* < 0.05) and non-reproductive proteins (median *d*_N_/*d*_S_ = 0.259; *p* < 0.05) for the branches to chimpanzee and Rhesus monkey. However, focusing on the same branches, Mann–Whitney *U*-test did not support different levels of *d*_N_/*d*_S_ in pre-mating sperm and non-reproductive proteins (*p* > 0.05; figure 2*b*; electronic supplementary material, table S13).

The chosen approach of comparing sequence evolution of functionally distinguished proteins within taxa made our results robust with respect to demographic effects. Hence, overall increased *d*_N_/*d*_S_ values in post-mating sperm proteins on the branches to multi-male breeders cannot be explained by demographic effects, such as population bottlenecks. In addition, 95% CIs of median *d*_N_/*d*_S_ values illustrated actually very similar evolutionary rates of pre-mating sperm and non-reproductive proteins on the branches to multi-male breeders and species with less intense post-mating sexual selection (figure 2; electronic supplementary material, table S12). This additionally argues against a general acceleration of sequence evolution on the branches representing multi-male breeders. Thus, increased levels of *d*_N_/*d*_S_ in post-mating sperm proteins on the branches to multi-male breeders most probably reflect that post-mating sexual selection is more effective in these species.

### (c) Inverse relationship between *d*_N_/*d*_S_ across codon sites and numbers of protein interaction partners

**Figure 3.**
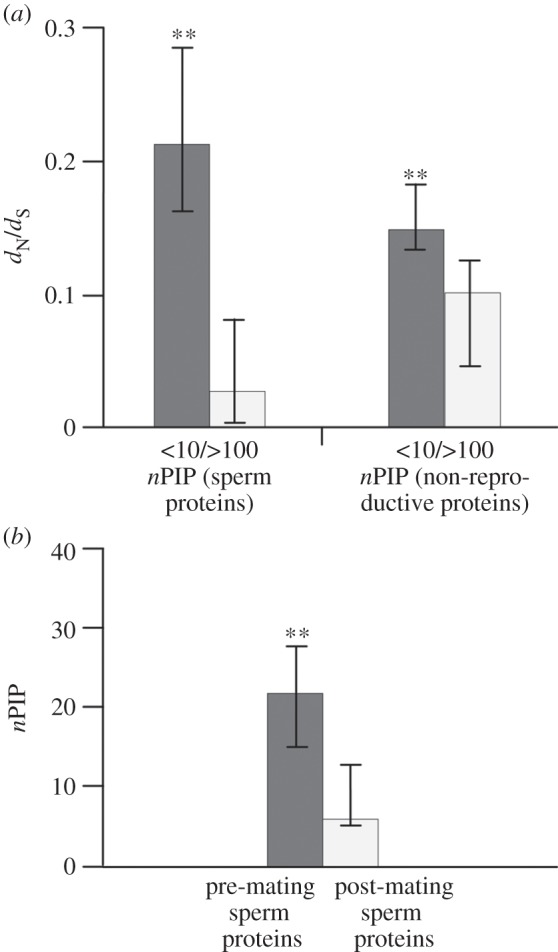
Sequence evolution and numbers of protein–protein interaction partners. (*a*) Levels of *d*_N_/*d*_S_ are significantly higher in proteins with less than 10 protein–protein interaction partners (*n*PIP < 10) than in proteins having more than 100 protein–protein interaction partners (*n*PIP > 100), irrespective whether taking sperm or non-reproductive proteins. Medians correspond to M8A estimates as inferred from eight primate orthologues per gene. (*b*) Pre-mating sperm proteins have significantly more protein–protein interaction partners than post-mating sperm proteins. Numbers of interacting proteins were derived from 17 databanks using the PSICQUIC meta-server. Vertical bars refer to 95% CIs inferred from 100 000 bootstrap replicates. Double asterisks (**) stand for significance at the 1% level (Mann–Whitney *U*-test) after sequential Bonferroni correction. See the electronic supplementary material, table S14 and figures S1 and S2.

Spearman's correlation indicated with high significance that *d*_N_/*d*_S_ values across sites (M8A) and *n*PIP per human orthologue were negatively correlated in non-reproductive proteins (*r*_s_ = −0.176; *p* < 0.01). In our sample of sperm proteins, the negative correlation was even more pronounced (*r*_s_ = −0.452; *p* < 0.01; electronic supplementary material, figures S1 and S2). In line with this general trend, levels of *d*_N_/*d*_S_ across sites (M8A) were consistently higher in sperm and non-reproductive proteins with *n*PIP < 10 (median *d*_N_/*d*_S_ = 0.213 and 0.149, respectively) than in their counterparts having *n*PIP > 100 (median *d*_N_/*d*_S_ = 0.027 and 0.101, respectively; *p* < 0.01, each; Mann–Whitney *U*-test; figure 3*a*; electronic supplementary material, tables S5, S6, S10 and S14). Thus, overall higher numbers of protein interactants in pre-mating (median *n*PIP = 22) than in post-mating sperm proteins (median *n*PIP = 6; *p* < 0.01; Mann–Whitney *U*-test) probably reflect increased levels of functional constraint in the former relative to the latter group (figure 3*b*; electronic supplementary material, table S14). Taken together, our data suggest that post-mating sperm proteins are less functionally constrained and more subjected to some form of post-mating sexual selection than are pre-mating sperm proteins. This pattern is more obvious in multi-male breeding species than in monogamous and unimale breeding species.

## 4. Discussion

### (a) Higher incidence of positive selection and elevated levels of *d*_N_/*d*_S_ across sites in post- versus pre-mating sperm proteins of primates

Proteins with germline-specific expression have repeatedly been described to evolve at higher evolutionary rates than proteins with expression maxima in other tissues [15,16,35]. This applies particularly to sperm proteins, such as sea urchin bindin [38], gastropod lysin [39] and members of our present sample, such as acrosin (ACR) and sperm autoantigenic protein 17 (SPA17) [26,40]. On the other hand, not all sperm proteins evolve rapidly and evolutionary rates actually depend on their detailed function [7,17,26]. Our observation of higher incidence of site-specific positive selection and overall increased *d*_N_/*d*_S_ values across sites in post-mating versus pre-mating sperm proteins confirms a general association between protein function and evolutionary rate (figure 1; see also the electronic supplementary material, tables S5 and S6). Hence, our data provide an additional example for an adaptive compartmentalization across the different steps of fertilization and, in particular, for an acceleration of sequence evolution towards sperm proteins involved in post-mating functions [7,17,18,41,42].

### (b) Numbers of protein–protein interactants suggest higher functional constraint in pre- versus post-mating sperm proteins

Protein–protein interactions mediate diverse intra- and intercellular processes and are pivotal for the functionality of cells and organisms. As impaired functioning of one protein affects all or at least some of its direct and indirect interactions, proteins having more interactants are more likely crucial for cell functioning than proteins with less interactants ([43]; see also [44,45]). Such increase in essentiality lowers non-synonymous substitution rates of proteins involved in more interactions: first, many initial amino acid exchanges require compensatory exchanges in binding partners to maintain pre-existing interactions [46]. Yet, each compensatory exchange is unlikely to occur within a tolerable time frame and, as a consequence, the domains mediating protein–protein interactions are usually highly conserved [47]. Second, interacting domains form larger portions of proteins having many than of those having few interactants [48]. This leads to stronger evolutionary conservation of total proteins with increasing numbers of interaction partners. In line with this, proteins at the centre of interaction networks have been shown to evolve at lower rates than peripheral proteins in a broad range of taxa [48–50]. Such negative association between numbers of interaction partners and substitution rates is exactly what we observed in our analyses of sperm and non-reproductive proteins. Especially, Spearman's rank correlation demonstrated that a protein evolves at lower rates the more interactions it is engaged in (see electronic supplementary material, figures S1 and S2). Contrasting proteins with few and many interacting partners (*n*PIP < 10 versus *n*PIP > 100) made the negative association between numbers of interactants and evolutionary rates even more obvious in our sperm and non-reproductive protein samples (figure 3*a*).

We are aware that the currently reported numbers of protein interactants are preliminary and that conclusions should be drawn with care. On the other hand, theoretical considerations suggest that the more protein interactants are known for a certain protein, the more additional interaction partners will be identified in the future [51]. Moreover, it is important to note that the present screen of *n*PIP data focused on protein–protein interactions within the male reproductive tract, and in particular on interactions within spermatozoa. Thus, lower numbers of protein interaction partners in post-mating sperm proteins (figure 3*b*) most likely reflect their peripheral role in the sperm interactome and not a bias from potentially less comprehensive data on postcopulatory interactions between male and female proteins. Consequently, we ascribe less incidence of site-specific positive selection and lower *d*_N_/*d*_S_ values across sites (M8A) in pre-mating versus post-mating sperm proteins to overall higher numbers of intracellular interactants in the former relative to the latter group (compare [52]).

### (c) Branch-specific *d*_N_/*d*_S_ values suggest most effective post-mating sexual selection in post-mating sperm proteins

While functional constraint generally counteracts non-synonymous substitutions, post-mating sexual selection is known to have an accelerating effect on sequence evolution of sperm proteins (e.g. [8–13]; see also [35,53]). Consistently, we observed significantly increased *d*_N_/*d*_S_ values in post-mating relative to pre-mating sperm and non-reproductive proteins for the branch to common chimpanzee and for foreground 2 comprising chimpanzee and Rhesus branches. On the contrary, post-mating sperm, pre-mating sperm and non-reproductive proteins evolved at similar rates on the gibbon branch and across foreground 1 which merges gibbon, human and gorilla branches (figure 2; electronic supplementary material, tables S12 and S13; for mating systems, see e.g. [27,28]). As outlined in the Results section, the statistical approach itself as well as similar *d*_N_/*d*_S_ estimates for pre-mating sperm and non-reproductive proteins for branches representing different matings systems makes it improbable that genome-wide effects biased our results. Rather, our data imply that some form of post-mating sexual selection, possibly sperm competition, accelerates sequence evolution of post-mating sperm proteins and that this phenomenon is more pronounced in species with increased female promiscuity.

Although these findings were reproduced in a two-species approach (gibbon versus chimpanzee) and in a multi-species approach (foreground 1 versus foreground 2), the increase of evolutionary rates of post-mating sperm proteins appeared not as strong in the combined analysis of chimpanzee and Rhesus branches as in the isolated analysis of the chimpanzee branch (see right panels in figure 2; electronic supplementary material, table S12). This might be partly owing to different samples of post-mating sperm proteins covered by both approaches (*n* = 25 and 43, respectively). The models employed (free-ratio model and branch model) might also have had an impact on the respective *d*_N_/*d*_S_ estimates. However, the most probable explanation for this observation is that levels of post-mating sexual selection are lower in Rhesus monkey (about three male periovulatory partners) than common chimpanzee (about eight male periovulatory partners; see [9]) despite a nominally identical mating system. Consequently, the inclusion of the Rhesus branch might have lowered *d*_N_/*d*_S_ estimates for post-mating sperm proteins.

It is further noteworthy that post-mating sperm proteins might also evolve at slightly increased evolutionary rates in northern white-cheeked gibbon, western lowland gorilla and human (see medians in figure 2, left panels). As loss-of-function mutations were not observed throughout the sampled cDNAs, neutrally evolving pseudogenes cannot explain this observation. Relaxed functional constraint, owing to strict monogamy as in pupal-mating butterflies [54], is also unlikely to explain the pattern. The finding might rather reflect occasional extra-pair and -group matings of females even in primate species with overall lower levels of post-mating sexual selection, as they frequently occur in white-tufted-ear marmoset and Sumatran orang-utan [29–32]. Still, the enhancement of evolutionary rates of post-mating sperm proteins was not significant in comparison with pre-mating sperm and non-reproductive proteins on the branches representing species with lower levels of post-mating sexual selection (figure 2).

## 5. Conclusion

Our data suggest that less functional constraint and more effective post-mating sexual selection explain overall increased non-synonymous/synonymous substitution rate ratios in post-mating relative to pre-mating sperm proteins. Present analyses further illustrate that the accelerating effect of post-mating sexual selection on sequence evolution is particularly effective on post-mating sperm proteins. But despite an apparent effect of functional constraint and post-mating sexual selection, other factors might affect sequence evolution of sperm proteins too. In particular, immune evasion, which describes the evolutionary escape of male reproductive proteins from female immune system, functional redundancy and defence against transposable elements through the piRNA pathway [55–57] may accelerate sequence evolution of sperm proteins. Expanding the focus on accelerating forces effective in female germline, other factors could be named, for example meiotic drive, which enhances sequence evolution of centromere and kinetochore proteins via competition for microtubule attachments in female meiosis [58]. Irrespectively of the latter, our data allow for some conclusions regarding the suitability of pre- and post-mating sperm proteins for applications in reproductive medicine and husbandry. Considering that higher numbers of interaction partners imply a central position in biological networks, and hence higher essentiality for their maintenance [59] and for cell functioning [43], pre-mating sperm proteins can be regarded as prime candidates for diagnosis and treatment of impaired male fertility. They may further be promising targets for the development of non-hormonal contraceptives, as illustrated by successful immunogenization against structural sperm proteins, for example sperm flagellar protein 2 (SFP2) [60]. Although selected post-mating sperm proteins may also be suitable targets for treatment of male infertility and non-hormonal contraception [61], strongest signatures of sexual selection suggest members of this category as the most promising targets for the identification of new biomarkers for male fertility levels in animal husbandry.
